# Three-dimensional propagation in near-field tomographic X-ray phase retrieval

**DOI:** 10.1107/S2053273315022469

**Published:** 2016-01-29

**Authors:** Aike Ruhlandt, Tim Salditt

**Affiliations:** a Institut für Röntgenphysik, Georg-August-Universität Göttingen, Friedrich-Hund-Platz 1, Göttingen, Germany

**Keywords:** tomography, phase retrieval, X-ray imaging

## Abstract

This paper presents an extension of phase retrieval algorithms for near-field X-ray (propagation) imaging to three dimensions, enhancing the quality of the reconstruction by exploiting previously unused three-dimensional consistency constraints. The approach is based on a novel three-dimensional propagator and is derived for the case of optically weak objects. It can be easily implemented in current phase retrieval architectures, is computationally efficient and reduces the need for restrictive prior assumptions, resulting in superior reconstruction quality.

## Abstract 2

An extension of phase retrieval algorithms for near-field X-ray (propagation) imaging to three dimensions is presented, enhancing the quality of the reconstruction by exploiting previously unused three-dimensional consistency constraints.

## 1. Introduction   

Over the last two decades the capabilities of X-ray tomography have been significantly improved by phase contrast methods. As opposed to conventional X-ray tomography, which is sensitive only to the amplitude (transmission) of the X-ray wave traversing an object, phase contrast techniques also take the phase shifting properties of the objects into account. This enables the visualization of weakly or non-absorbing soft tissues in biomedical imaging, or nanoscale structures in material science. Phase contrast is easily achieved without additional optical elements by free space propagation. The associated self interference of the object’s exit wave over the controllable distance Δ between object and detector (Wilkins *et al.*, 1996 ▸; Cloetens *et al.*, 1999 ▸; Paganin & Nugent, 1998 ▸) encodes phase information into measurable intensities. The standard approach in evaluating phase contrast tomography data is a two-step reconstruction procedure. First, the phase retrieval is carried out, *i.e.* the complex valued exit wave is retrieved from the intensity measurements, separately for each projection angle. Secondly, all projections are combined to a three-dimensional volume using inverse Radon transform methods, in most cases a filtered back-projection (FBP). This is in sharp contrast to far-field coherent diffractive imaging, where phase retrieval is performed not only on projections, but commonly directly in three dimensions (Miao *et al.*, 2001 ▸, 2005 ▸; Chapman *et al.*, 2006 ▸).

The phase retrieval step is considered to be the main challenge. Considerable efforts have concentrated on phase contrast algorithms beyond the simple but flawed holographic reconstruction by numerical back-propagation (Paganin, 2006 ▸). Deterministic but approximative solutions have been formulated based on the transport-of-intensity equation (TIE) (Paganin *et al.*, 2002 ▸; Groso *et al.*, 2006 ▸; Bronnikov, 1999 ▸), or on the analytic form of the free space contrast transfer functions (CTF) (Cloetens *et al.*, 1999 ▸; Turner *et al.*, 2004 ▸; Gureyev *et al.*, 2004 ▸; Langer *et al.*, 2012 ▸; Hofmann *et al.*, 2011 ▸). The general strategy to overcome reconstruction artifacts such as the well known twin-image problem is to either use data sets with more than a single object-to-detector distance (Cloetens *et al.*, 1999 ▸; Allen & Oxley, 2001 ▸; Latychevskaia & Fink, 2007 ▸; Hong *et al.*, 2012 ▸; Krenkel *et al.*, 2013 ▸), restrictive prior information such as known relationships between phase shift and absorption (Paganin, 2006 ▸; Wu *et al.*, 2005 ▸) or known compact support of the object (Gerchberg & Saxton, 1972 ▸; Giewekemeyer *et al.*, 2011 ▸; Bartels *et al.*, 2012 ▸). The constraints due to prior information are usually implemented by iterative projection algorithms.

To overcome these limitations and provide a solution in particular for tomography scans at a single detection distance Δ, which is the most relevant case in practice, we and others have recently proposed a coupling of the two steps, phase retrieval and tomographic reconstruction (Kostenko *et al.*, 2013 ▸; Ruhlandt *et al.*, 2014 ▸). We could show that a combined phase retrieval and algebraic tomographic reconstruction scheme termed ‘iterative reprojection phase retrieval’ (IRP) enhances the reconstruction quality and allows one to retrieve the phase of objects of mixed composition without the need for additional *a priori* knowledge (Ruhlandt *et al.*, 2014 ▸). In particular, IRP was found to stabilize the reconstruction of low spatial frequencies which have previously hampered single-distance phase retrieval. This differs from combinations of the phase retrieval and FBP published before, which were implemented not to achieve coupling, but enhanced speed (Gureyev *et al.*, 2004 ▸; Bronnikov, 2002 ▸). We attribute the enhancements in quality to the tomographic consistency condition (Helgason, 1965 ▸; Ludwig, 1966 ▸), which states that the different projections of an object are not independent from each other. This has been found very useful before in conventional absorption tomography, for example in the reconstruction of incomplete data (missing wedge, Kudo & Saito, 1991 ▸). Unfortunately, the gain in performance by IRP came at the cost of substantial computational effort, as well known also from previous algebraic tomographic reconstruction (ART) for the case of conventional absorption CT (computed tomography) (Gordon *et al.*, 1970 ▸; Kak & Slaney, 1988 ▸). Hence, the combined phase retrieval and ART approach could so far be applied only to small data volumes.

In this work, we show that phase retrieval for optically weak objects can be enhanced by an inversion of the two steps, *i.e.* by first performing the inverse Radon transform, followed by a computationally efficient three-dimensional phase retrieval. The first step can be implemented for example by a fast FBP, the second by three-dimensional fast Fourier transformations (FFT). This approach has been used before in the context of deterministic one-step phase retrieval (Frank & Penczec, 1995 ▸; Cloetens *et al.*, 1997 ▸). In this work we show that in combination with iterative phase retrieval, this three-dimensional propagation scheme exhibits superior reconstruction quality. Furthermore, propagation of entire three-dimensional objects sheds new light on the nature of the three-dimensional phase contrast tomography problem. In this article, first the theoretical concept is introduced, followed by a numerical implementation demonstrating the capabilities of the approach.

## 2. Theory   

**Figure 1:**
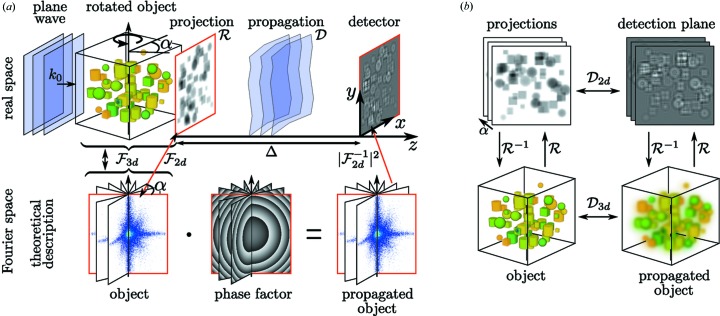
(*a*) Schematic of holographic phase contrast imaging. A plane wave illuminates the object, leading to an exit wave in the *xy* plane as given by the projected optical indices, followed by free space propagation resulting in holographic phase contrast formation as recorded in the detection plane at distance Δ. The projection 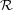 and propagation 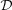 process can be described theoretically in Fourier space by multiplying the central slices of the object’s Fourier transform with a radially symmetric phase factor. (*b*) For weakly interacting objects, the projection operation 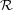 and the propagation operation 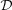 can be permuted, allowing for the propagation of the three-dimensional index of refraction.

In X-ray imaging, the interaction of the radiation with an object is usually described by a three-dimensional index of refraction, 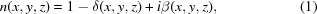
where 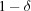 causes the phase shift and the imaginary part 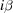 the absorption in the object for a given wavelength λ. Notice that we consider monochromatic radiation. The illumination wave is treated as a plane wave 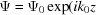) propagating along the optical axis *z* with the wavenumber 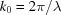. For a homogeneous slab of thickness *t* the phase shift induced by the object with respect to the propagation in free space is simply given by 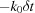, and the wave amplitude by 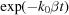. In most practical applications of hard X-ray coherent imaging, the object is weak enough that the projection approximation holds (Thibault, 2007 ▸) and the propagation of the wave within the sample can be neglected. The distribution of the index 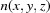 then leads to a spatially modulated *exit wave* 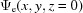 in the plane 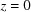 directly behind the object, which is determined only by the *projection* 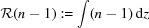 of the index of refraction along the optical axis: 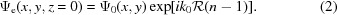
Further, for optically weak objects 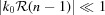 the exponential function can be linearized to 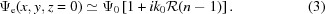
The propagation 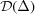 of the exit wave 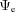 in free space can be expressed by a multiplication in Fourier space. For a given propagation distance Δ the two-dimensional (2d) Fourier transform 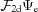 is multiplied point-wise with the radially symmetric *propagator* 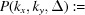 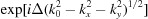 (Fourier space coordinates 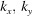) followed by a Fourier back-transform 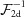 (Paganin, 2006 ▸): 
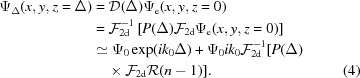
In the last step, the weak object approximation has been used. According to the Fourier slice theorem, the two-dimensional Fourier transform of a projection is identical to the central slice of the three-dimensional (3d) Fourier transform of the index of refraction 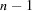, normal to the direction of the projection: 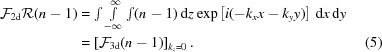
Hence, every operation carried out in the two-dimensional Fourier space of the projection, notably the multiplication with the propagator 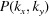, can equally be applied to the corresponding central slice in the three-dimensional (3d) Fourier space. In the linearized approximation of optically weak objects this allows one to invert the order of the projection and propagation: 
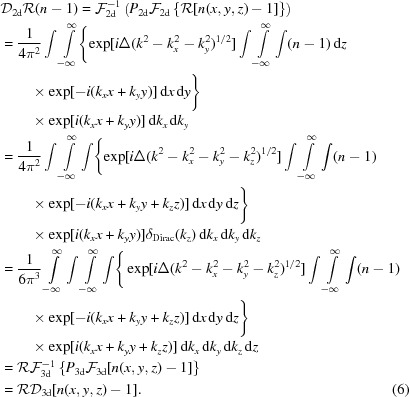
To avoid misunderstandings, we note that 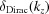 in the expression above denotes the Dirac delta distribution and not the dispersive decrement of the index of refraction. Propagating the three-dimensional object (volume) first and projecting subsequently leads to the same result as conventional two-dimensional propagation of a projection. Moreover, this holds for every projection angle. For tomography, the object is rotated around the *y* axis, as shown in Fig. 1 ▸(*a*), and projected under a large number of angles 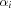. In Fourier space, these projections correspond to planes with normal vectors in the *xz* plane sharing one common axis 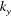 corresponding to the axis of rotation, as also illustrated in Fig. 1 ▸(*a*). Propagating all the projections simultaneously about a distance Δ is equivalent to multiplying 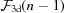 point-wise with the phase function 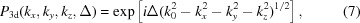
which appeared in equation (6) as the three-dimensional generalization of the conventional two-dimensional propagation kernel 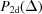. Again, 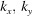 and 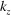 are the components of the sample’s Fourier space and not the components of the wavevector 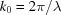. Hence, the three-dimensional propagator 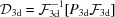 describes a method to propagate the entire three-dimensional object in the near field and is the most important concept and tool in this work. We denote the output as ‘propagated object’. While we only use the above formulation of the propagator in this work, the method is not restricted to this particular choice. Alternative formulations including the paraxial approximation of equation (7) (Paganin, 2006 ▸) or of the Rayleigh–Sommerfeld/Fresnel–Kirchhoff diffraction integrals (Voelz & Roggemann, 2009 ▸) could also be used. The three-dimensional nature of the ‘propagated object’, and the fact that the propagation between object and detection planes is now carried out for the entire three-dimensional volume, has some important consequences and is conceptually different from the conventional propagation of two-dimensional wavefronts. One interesting aspect of the three-dimensional propagator approach is given by the fact that phase retrieval can be performed on central planes through the object with arbitrary orientation, including in particular planes orthogonal to the tomographic rotation axis. In view of computational speed, we found that the single three-dimensional propagation outperformed the conventional *N* two-dimensional propagations, in particular for data sets with a high number of projections *N*.

Next, we show how the three-dimensional propagator can be used advantageously in iterative phase retrieval. To this end, we do not restrict ourselves to small propagation distances, *i.e.* validity of the TIE regime (Paganin, 2006 ▸), but consider the problem for general Fresnel numbers including the holographic regime. We remain, however, within the weak object approximation, as stated above. Note that, strictly speaking, the linearization with respect to the object needed here is valid beyond the strict weak phase object [see for example the slowly varying phase condition introduced in Guigay (1977 ▸) and Gureyev & Nesterets (2015 ▸)]. Since in an experiment only the intensity 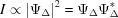 of the propagated exit field can be measured, we do not have direct access to the propagated three-dimensional object. However, we can apply an inverse Radon transform 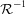 on the intensities 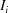, combining all 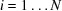 projections to a three-dimensional ‘intensity field’ 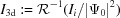. Using equation (6), the usual decomposition of the measured intensities into the different components of the hologram is given as 
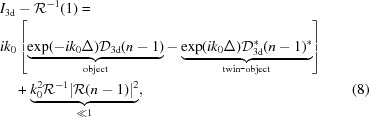
formulating the well known twin-image problem of holography in three dimensions. The expression shows that the three-dimensional ‘intensity field’ 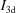 is *consistent*, since it can be written as the sum of the propagated object and the complex conjugated ‘twin object’ 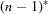 propagated about the distance 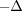, since 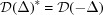. Tomographic consistency, described in detail by Helgason and Ludwig (Helgason, 1965 ▸; Ludwig, 1966 ▸; Kudo & Saito, 1991 ▸), states that projections from the same object are not independent from each other. The Fourier slice theorem already shows that all projections share a common intersection line in Fourier space. For objects of finite size, the central slices in Fourier space can be seen as broadened by a convolution with the object’s support. This introduces a dependence between all slices. Explicitly, one cannot change the value at any given point in Fourier space without violating consistency for an object of finite size. From equation (8) we can infer that the twin-object/phase problem does not introduce such tomographical inconsistencies. In return, this property cannot be exploited to improve phase retrieval directly. In particular, we expect only limited improvements for three-dimensional phase retrieval by deterministic one-step algorithms like a direct CTF inversion, where the three-dimensional inversion has been used previously (Frank & Penczec, 1995 ▸; Cloetens *et al.*, 1997 ▸) if carried out in three dimensions rather than two dimensions, even if the geometry of the three-dimensional problem is different from two-dimensional imaging. With the definition 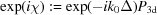 and 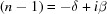, equation (8) is rewritten in Fourier space as 
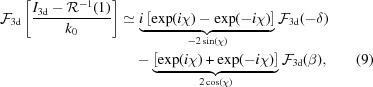
illustrating the structure of the CTF in three dimensions. The CTF_3d_ with zero values on spherical shells does not contain all information about the object. Therefore, one has to resort to further constraints in phase retrieval, *i.e. a priori* information, such as positivity, range restrictions of the object functions, or known support of the object. However, we have shown previously that the performance of iterative phase retrieval with constraints such as positivity can be dramatically improved if tomographic consistency is enforced (Ruhlandt *et al.*, 2014 ▸). If the phase retrieval is carried out in three dimensions according to the concept presented, consistency is guaranteed automatically.

## 3. Simulation and numerical results   

**Figure 2:**
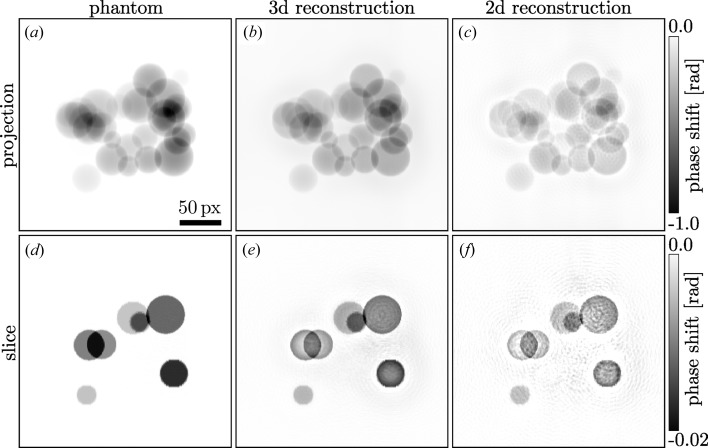
Comparison of the phase shifts reconstructed using the conventional two-dimensional propagation and the three-dimensional propagation. The upper row shows a typical projection of the phantom/reconstructed volume, the lower row shows an *x*–*z* slice, normal to the axis of rotation of the volume. In the left column the original phantom is depicted, the central column contains the results of the three-dimensional propagation method presented here and the right column shows the conventional results. The scale bar is the same for all images; the colour bars apply to the corresponding rows.

To validate and to illustrate the concept introduced above, we show an exemplary simulation. A sample was designed consisting of 30 spheres with a diameter between 20 and 50 px (px stands for pixel) distributed randomly on a 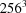 voxel grid. For each sphere, a combination of 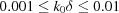 and 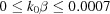 was selected randomly, resulting in a maximum phase shift of 1.5 rad and a maximum absorption of 6% in the projections.^1^ The object was projected to 402 equidistant angles in the range 0° 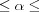 180° to satisfy the angular sampling criterion. To simulate the intensity data 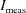 in the detection plane, all projections were propagated about a distance 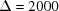
 px with a wavelength of 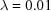
 px, resulting in a Fresnel number of 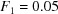 for one pixel. This choice corresponds for example to a relevant experimental setting in X-ray phase contrast tomography with photon energy of 12.4 keV, an effective pixel size of 10 nm and an effective sample-to-detector distance of 20 µm. The phase retrieval algorithm cycles between the object plane and detection plane, always propagating the wavefield Φ. In the detection plane, the modulus constraint 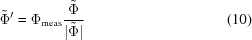
is applied, setting the modulus of the wavefield 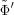 to the measured values 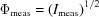 while keeping the phase information. In the object plane we demand positivity for both the δ and β parts of the index of refraction which follows directly from its definition and the projection approximation: 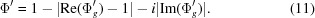
Additionally, we enforced 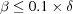, which is justified for a large class of hard X-ray experiments and samples, including in particular biological tissues. This constraint is implemented in the following way: we first set 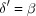 in pixels where 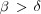 and only afterwards set 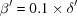 where 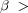 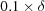. 1000 iterations of this ‘soft coupling’ scheme were carried out for (i) the conventional method with the phase information of each detector image retrieved individually followed by a filtered back-projection (‘two-dimensional reconstruction’) and (ii) for the three-dimensional volume starting with a filtered back-projection of the 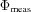. Typical results are depicted in Fig. 2 ▸, showing clearly the better quality of the three-dimensional approach. In the supporting information for this paper, additional simulation results are presented for noisy intensity data (Poissonian noise corresponding to 10 000 ph px^−1^; ph stands for photon) as well as results without the ‘soft coupling’ and thus without any assumption on the object. While the overall quality of these reconstructions is not as good as in Fig. 2 ▸, both cases still show a major improvement of the three-dimensional approach compared to the two-dimensional reconstruction and illustrate the robustness of the method. The entire three-dimensional phase retrieval process was carried out within about 2 min using MATLAB and an NVIDIA GTX Titan GPU. Example code in MATLAB/Octave for the simulation and the explained three-dimensional reconstruction is available as supporting information.

**Figure 3:**
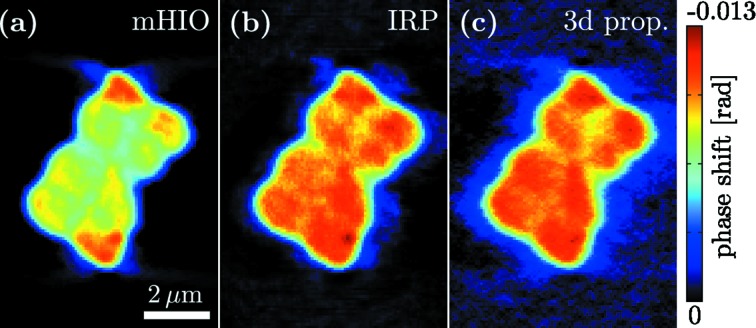
Comparison of different reconstructions from the same experimental data. All images show the same slice through the reconstructed three-dimensional volumes of a *Deinococcus radiodurans* bacteria. (*a*) illustrates the ‘conventional’ mHIO reconstruction reported before (Bartels *et al.*, 2012 ▸; Ruhlandt *et al.*, 2014 ▸) where additional support constraints have been used. (*b*) The IRP result of the same data shows a much more homogeneous signal distribution without the use of support information. The method described here leads to the result depicted in (*c*) showing a comparable reconstruction quality as IRP but calculated in a fraction of the time.

Based on the simulations, we can expect that the approach also performs well on noisy experimental data. To this end, we have tested the algorithm on the data presented previously in Bartels *et al.* (2012 ▸) and Ruhlandt *et al.* (2014 ▸), corresponding to holographic projection images of freeze-dried *Deinococcus radiodurans* bacteria, dispersed on ultra-thin Si_3_N_4_ membranes and recorded using 13.8 keV radiation exiting from a waveguide [see Bartels *et al.* (2012 ▸) for experimental details]. The tomographic scan (single defocus distance data set) comprised 83 projection angles distributed over 162°. The reconstructed phase of the bacteria as resulting from the three-dimensional propagator approach in combination with ART is displayed for a representative slice through the object (Fig. 3 ▸). For comparison, reconstructions are shown as obtained by the previously presented algorithms, the modified HIO (mHIO) (Giewekemeyer *et al.*, 2011 ▸; Bartels *et al.*, 2012 ▸) and the IRP (Ruhlandt *et al.*, 2014 ▸). A major difference between mHIO on the one side, and IRP and the scheme reported here (three-dimensional propagation) on the other is that mHIO needs additional support constraints for phase retrieval, while the latter do not. Note that, despite this additional constraint, mHIO leads to artifacts such as the increase of density towards the top and bottom corners, while the reconstruction of IRP and the method presented here yield a much more plausible density distribution. This advantage of IRP over mHIO has been stressed before (Ruhlandt *et al.*, 2014 ▸), but came at the expense of significant numerical complexity, while the three-dimensional propagation reaches a quality comparable to IRP in a fraction of the computation time.

## 4. Summary and conclusions   

While three-dimensional reconstruction in far-field diffraction has long been known to aid phase retrieval (Marchesini *et al.*, 2003 ▸), the generalization to the near-field case was less obvious since the measurements (near-field patterns) cannot in general be considered to be sub-manifolds of a three-dimensional reciprocal space of the sample. Therefore, an algebraic tomography scheme was previously proposed for the purpose of three-dimensional reconstruction of arbitrary objects (Kostenko *et al.*, 2013 ▸; Ruhlandt *et al.*, 2014 ▸), however at the cost of numerous iterations of performing Radon and inverse Radon transforms.

In this work, we have introduced a new approach to near-field tomographic phase retrieval in the limit of an optically weak object. The usual sequence of first retrieving the phase information of all projections individually followed by an inverse Radon transform is inverted. A three-dimensional volume is computed from the measurements, followed by iterative propagations and the application of constraints in three dimensions. Since the inverse Radon transform is performed only once, a tremendous enhancement in reconstruction speed is obtained with respect to previous combined schemes of iterative phase retrieval and tomographic reconstructions. At the same time, the three-dimensional propagation method preserves the essential advantages of tomographic consistency, which is intrinsically enforced by the three-dimensional scheme and was found to stabilize phase retrieval with otherwise under-determined data.

Even though the three-dimensional propagation does not lead to major improvements in the quality of direct CTF phase retrieval, it can be useful to investigate the influence of regularization parameters directly in three dimensions.

Another immediate advantage of the three-dimensional scheme not yet exploited here is directly obvious: in three dimensions, the formulation of entirely new and more powerful constraints is possible, since they can be applied directly on the object rather than its projections. This applies for example to a much more accurate and constraining support determination, to positivity as well as to sparsity or to regularization procedures. In general, constraints can not only be formulated in physically correct and direct terms in three dimensions, but can possibly also be applied to a higher fraction of voxels. Finally, the measurement scheme could easily be generalized to several detection planes, and – somewhat less straightforward – also to more complex illumination wavefields than plane waves.

The theory presented here is based on the assumption of an optically weak object. However, better phase retrieval results were obtained in three dimensions even if the maximum phase shift in some projections was as high as 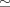 1.5 rad, as shown in the example of Fig. 2 ▸.

In future work, extensions to optically thick samples could be investigated. The corresponding exit fields of a sample may be expected to fulfil generalized consistency criteria which might be exploitable, and problems of phase wrapping may also be treated much better if the object is represented in three dimensions throughout the phase retrieval process.

More straightforward extensions of the present work will extend the numerical comparison between two-dimensional and three-dimensional phase retrieval and establish how the reconstruction quality gain depends on all experimental parameters, including number of projections, Fresnel number, added noise or even systematically perturbed data. To this end, the MATLAB example code (available as supporting information) may be helpful.

## Supplementary Material

Supplementary figures. DOI: 10.1107/S2053273315022469/mq5036sup1.pdf

Example code in MATLAB/Octave. DOI: 10.1107/S2053273315022469/mq5036sup2.txt

## Footnotes

1 This value clearly violates the assumption of a weakly interacting object, but illustrates that the advantage of the three dimensions persists even to larger phase shifts.
