# Supplementary material for: Three-dimensional propagation in near-field tomographic X-ray phase retrieval
Source: Acta Crystallogr A Found Adv. 2016 Jan 29;72(Pt 2):215–21. doi: 10.1107/S2053273315022469 (PMC4770872; doi:10.1107/S2053273315022469)
Supplement: Supplementary file 1 [file a-72-00215-sup1.pdf]

Three-dimensional propagation in near-field tomographic x-ray phase retrieval: Supporting Online Material

AIKE RUHLANDT* AND TIM SALDITT

Institut für Röntgenphysik, Georg-August-Universität Göttingen,

Friedrich-Hund-Platz 1, Göttingen, Germany. E-mail: aruhlan@gwdg.de

While Fig. 2 in the main article compares the reconstructed phase shift with the ground truth, Fig. 1 in this Supporting Online Material depicts the corresponding absorption values of the same reconstruction.

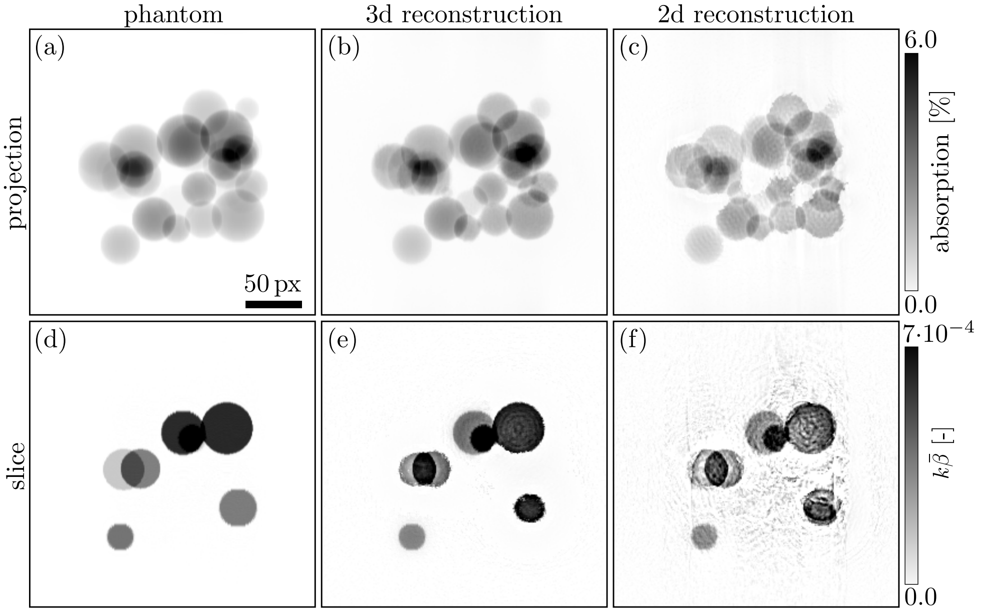

Fig. 1. Comparison of the absorptions reconstructed using the conventional 2d propagation and the 3d propagation. The upper row shows a typical projection of the phantom / reconstructed volume, the lower row shows a central $x - z$-slice (depth of one pixel), normal to the axis of rotation of the volume. In the left column the original phantom is depicted, the central column contains the results of the 3d propagation and the right column shows the conventional results. The scale bar is the same for all images, the colorbars apply to the corresponding rows.

The influence of noise is shown in Fig. 2 and 3, where Poissonian noise corresponding to a mean count rate of 10000 photons per pixel was added to the simulated intensities in the detection plane. The most obvious difference is a nearly homogenious background in the reconstructed phase shifts using the three dimensional propagation. This offset introduced by the noise is generally allowed for the reconstructed phases considering our constraints. The general advantage of the new scheme presented here persists.

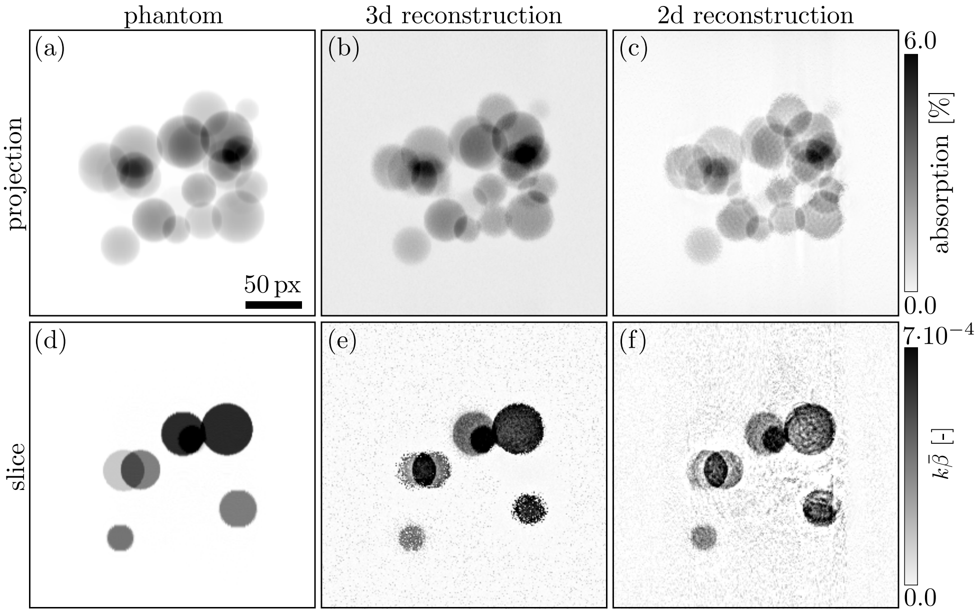

Fig. 2. Comparison of the phase shifts reconstructed from noisy data using the conventional 2d propagation and the 3d propagation. The upper row shows a typical projection of the phantom / reconstructed volume, the lower row shows a central $x - z$-slice, normal to the axis of rotation of the volume. In the left column the original phantom is depicted, the central column contains the results of the 3d propagation and the right column shows the conventional results. The scale bar is the same for all images, the colorbars apply to the corresponding rows.

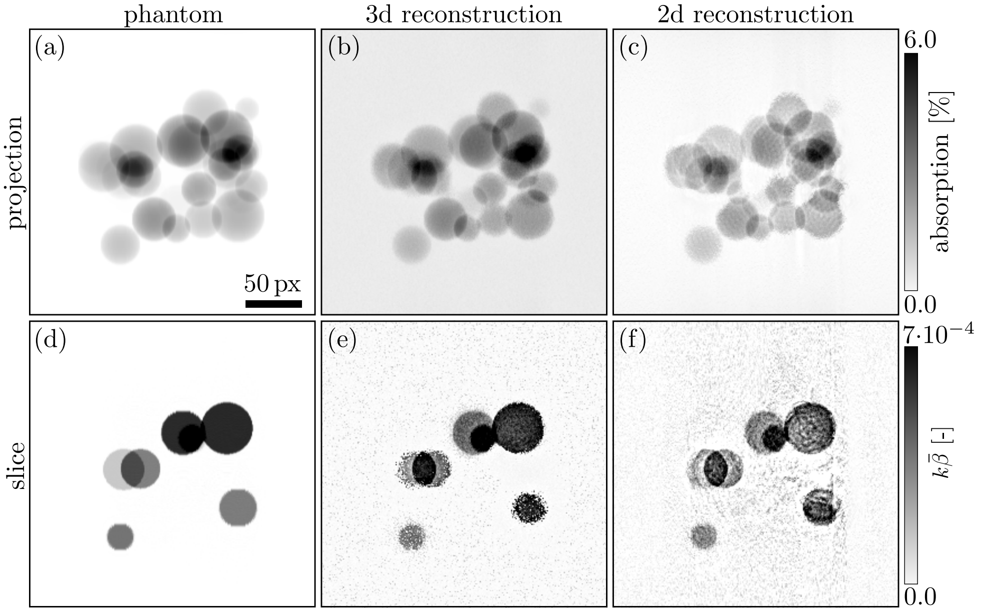

Fig. 3. Comparison of the absorptions reconstructed from noisy data using the conventional 2d propagation and the 3d propagation. The upper row shows a typical projection of the phantom / reconstructed volume, the lower row shows a central $x-z$-slice, normal to the axis of rotation of the volume. In the left column the original phantom is depicted, the central column contains the results of the 3d propagation and the right column shows the conventional results. The scale bar is the same for all images, the colorbars apply to the corresponding rows.

If the 'soft coupling' $0 \leq \beta \leq \kappa \cdot \delta$ is relaxed from $\kappa = 0.1$ to $\kappa = 1$, the simulation shows the results as depicted in Fig. 4 and 5. Again, Poissonian noise corresponding to a mean count rate of 10000 photons per pixel was added to the simulated intensities and the results are shown in the lower right half of the corresponding images.

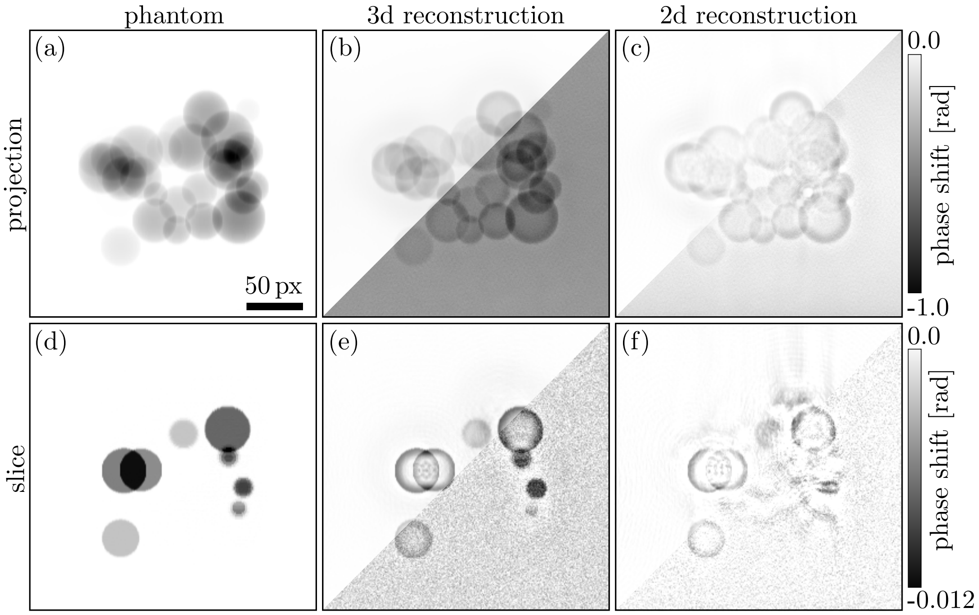

Fig. 4. Comparison of the phase shifts reconstructed using the conventional 2d propagation and the 3d propagation without restrictive assumptions. The upper row shows a typical projection of the phantom / reconstructed volume, the lower row shows a central $x-z$-slice, normal to the axis of rotation of the volume. In the left column the original phantom is depicted, the central column contains the results of the 3d propagation and the right column shows the conventional results. For all reconstructions, the lower right half shows the result for noisy data as described in the text. The scale bar is the same for all images, the colorbars apply to the corresponding rows.

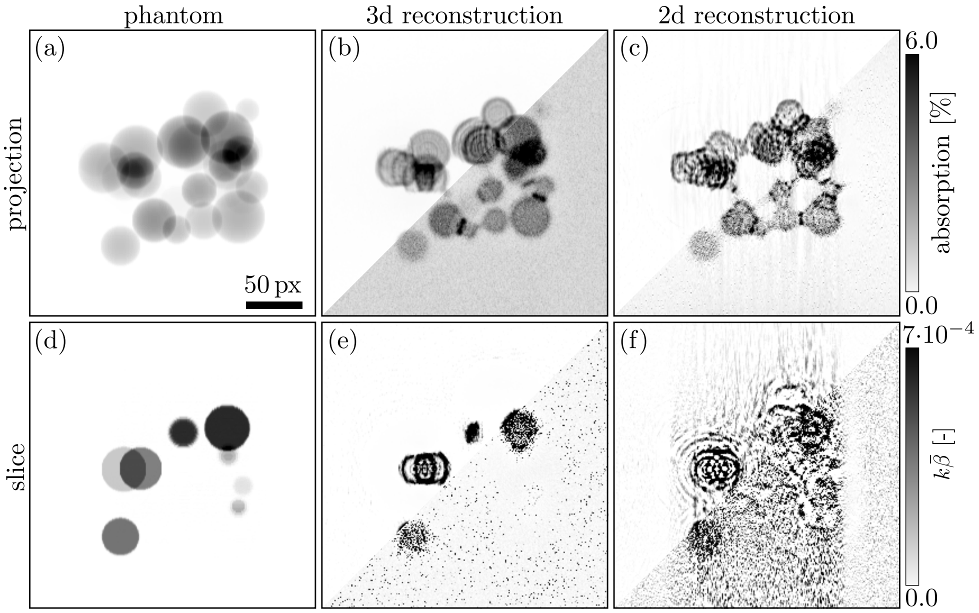

Fig. 5. Comparison of the absorptions reconstructed using the conventional 2d propagation and the 3d propagation. The upper row shows a typical projection of the phantom / reconstructed volume, the lower row shows a central $x-z$-slice, normal to the axis of rotation of the volume. In the left column the original phantom is depicted, the central column contains the results of the 3d propagation and the right column shows the conventional results. For all reconstructions, the lower right half shows the result for noisy data as described in the text. The scale bar is the same for all images, the colorbars apply to the corresponding rows.

The implementation details are summarized in the attached Matlab/Octave file which easily allows to reproduce all results shown here.
